# Adaptability and nutritional analysis of a newly isolated *Chlorella* sp. NeZha in brackish and marine environments with potential bioeconomic impacts

**DOI:** 10.3389/fnut.2024.1460675

**Published:** 2024-08-14

**Authors:** Shuai Yuan, Ming Du, Xianhui Li, Ke Xu, Kaining Zhang, Xiaoya Liu, Jiangxin Wang

**Affiliations:** ^1^School of Life Sciences and Oceanography, Shenzhen University, Shenzhen, China; ^2^Hainan Chenhai Aquatic Co., Ltd., Sanya City, Hainan, China; ^3^PingYi County Hospital of Traditional Chinese Medicine, LinYi, Shangdong, China

**Keywords:** *Chlorella* sp., aquaculture, chlorophyll a, adaptability, salinity

## Abstract

**Introduction:**

The microalga Chlorella sp. NeZha, recently isolated from a balcony environment, shows significant adaptability across various salinity conditions, including seawater (SeaW), freshwater (FreshW), and high salinity levels (45‰). This study investigates its potential for sustainable aquaculture and biotechnological applications.

**Methods:**

Morphological and genetic identification were conducted using optical microscopy and DNA sequencing. The microalga was cultivated in a 400 L outdoor photobioreactor, and its biochemical composition, including chlorophyll a, carbohydrate, protein, and lipid content, was analyzed. Its compatibility with zooplankton and growth in aquaculture wastewater were also evaluated.

**Results:**

Chlorella sp. NeZha produced chlorophyll a at concentrations exceeding seaweed and Spirulina by 10- and 5-fold, respectively, with a dry weight chlorophyll a content of 34.25 mg/g and 25 pg./cell. The microalga also contained carbohydrate (~33%), protein (~20%), and lipids (~14%). It was compatible with zooplankton species, such as rotifers and brine shrimp, and showed promising growth in aquaculture wastewater.

**Discussion:**

The findings suggest that Chlorella sp. NeZha is a viable candidate for sustainable aquaculture and biotechnological applications, offering high nutritional value and environmental resilience. Its adaptability to diverse salinity conditions and ability to thrive in wastewater highlight its potential for bioremediation and use as feedstock for zooplankton. Further research is recommended to optimize its cultivation and explore broader applications.

## 1 Introduction

Microalgae are increasingly recognized for their potential in sustainable biotechnological applications, including biofuel production and environmental bioremediation (1, 2). The ability of microalgae to adapt to environmental fluctuations, particularly changes in salinity, is crucial for their survival and optimal productivity. *Chlorella*, a genus of green microalgae, has garnered significant attention due to its high photosynthetic efficiency and nutrient-dense profile. Among its species, *Chlorella vulgaris* stands out for its remarkable nutrient composition, making it a favored dietary supplement (3, 4). It is a complete protein source, providing all essential amino acids, and is abundant in vitamins and minerals, including vitamin C, B12, iron, magnesium, and calcium. Additionally, *Chlorella* is loaded with antioxidants like beta-carotene, which help reduce oxidative stress and prevent chronic diseases (5).

Incorporating *Chlorella* into the diet is associated with multiple health benefits. Its rich nutrient content supports enhanced immune function and healthy digestion. The presence of vitamins and minerals contributes to overall well-being, while its antioxidant properties offer potential protective effects against various chronic conditions (6–9). Also, microalgae, including *Chlorella* species, are also indispensable food sources or nutrient supplements for secondary live prey, including rotifers, *Artemia*, and copepods (3, 10–15).

Chlorophyll a (Chl a) content in microalgae is a critical indicator of their photosynthetic capacity and is influenced by various environmental factors. Studies have shown that nutrient availability, temperature, light intensity, and culture conditions significantly impact Chl a levels. For instance, nitrogen and sulfur deprivation reduce Chl a content in *Chlamydomonas reinhardtii* with a range of 0.03–0.035 pg./cell (16, 17). Temperature and irradiance have synergistic effects, influencing the biochemical composition of microalgae such as *Pavlova lutheri*, which exhibited 0.47 pg./cell Chl a content under varying conditions (18). Additionally, culture conditions, including mixotrophic environments, affect Chl a content in species like *Cheatoceros* sp., which ranged from 15.49 to 23.65 mg/g (19). Phosphorus levels and temperature also play crucial roles, impacting Chl a in *Scenedesmus obliquus*, which showed 16.23 mg/g under different phosphorus concentrations and temperatures (20). Light intensity and nutrient stress conditions further modulate Chl a content, highlighting the importance of optimizing environmental parameters for maximizing chlorophyll production in microalgae (21–24). These findings underscore the complex interplay of factors affecting Chl a content, which is essential for enhancing the productivity of microalgal biomass for biotechnological applications.

High Chl a content in microalgae has significant advantages in various fields, including health, biotechnology, and environmental applications. Nutritionally, Chl a provides essential vitamins and antioxidants, aiding in detoxification and immune system enhancement, making microalgae a popular choice for dietary supplements and health foods (25–27). In terms of photosynthetic efficiency, Chl a is crucial for converting light energy into chemical energy, which can lead to faster growth rates and higher biomass yields, benefiting large-scale algal cultivation for biofuels and other biomass-derived products (5, 28). Biotechnologically, microalgae with high chlorophyll levels are valuable for biofuel production due to their enhanced solar energy conversion capabilities, improving the economic viability of sustainable energy solutions (29). Environmentally, these microalgae play a key role in bioremediation, helping to remove pollutants and reduce CO_2_ levels, thereby contributing to climate change mitigation. Additionally, chlorophyll from microalgae is used in cosmetic and pharmaceutical products for its anti-inflammatory and wound-healing properties, promoting skin health and being incorporated into creams, ointments, and other topical applications (25–27). These multifaceted benefits underscore the importance of researching and cultivating microalgae with high Chl a content for health, energy, and environmental sustainability.

*Chlorella* sp. NeZha, identified through isolation in a balcony environment, exhibits unique properties that distinguish it from other *Chlorella* species. Notably, *Chlorella* sp. NeZha demonstrates exceptional robustness under varying salt conditions, making it a promising candidate for applications in both saline and freshwater environments. This adaptability not only enhances its potential for large-scale cultivation but also broadens its utility across diverse biotechnological fields. The unique characteristics of *Chlorella* sp. NeZha, including its high chlorophyll content and resilience to environmental stressors, make it an excellent subject for further research and development aimed at maximizing the benefits of microalgal applications in health, biotechnology, and environmental sustainability. This study aims to elucidate the specific advantages of *Chlorella* sp. NeZha in these contexts, providing a foundation for its future utilization and highlighting its contributions to advancing microalgal biotechnology.

Specifically, our research aims to evaluate the photosynthetic efficiency, nutrient composition, and bioremediation capabilities of *Chlorella* sp. NeZha under varying environmental conditions. We hypothesize that *Chlorella* sp. NeZha will exhibit superior adaptability and productivity compared to other *Chlorella* species, making it an ideal candidate for biotechnological applications.

## 2 Materials and methods

### 2.1 Isolation and purification of microalgal strains

The microalgal strain *Chlorella* sp. NeZha was isolated from a residential balcony located on Hainan Island, China, an area known for its stable climatic conditions with monthly average air temperatures ranging between 22.5 and 25.6°C and monthly solar radiation levels varying from 375 to 483 MJ/m^2^. Following its isolation, the *Chlorella* sp. NeZha strain was subjected to a purification process utilizing established microbiological techniques (30). Initial screenings for viable strains were conducted at ambient room temperature. This involved culturing on BG11 medium agar plates under constant exposure to fluorescent lighting providing a photon flux density of 100 μmol/m^2^/s. From these screenings, six strains exhibiting robust growth were further propagated under identical conditions in shake flask batch cultures. These cultures were maintained in 250 mL Erlenmeyer flasks, each containing 100 mL of working culture volume and agitated at a rate of 130 rpm. The screening period for each culture spanned 20 days, conducted under aseptic conditions to prevent contamination. At the conclusion of the screening phase, the sterility of each culture was verified by additional plating on BG11 medium agar plates to ensure the absence of contaminant organisms. Morphological analysis was conducted using optical microscopy, and genetic identification was verified through DNA sequencing based on standard genomic DNA extraction and PCR sequencing for 18S rDNA and ITS (Supplementary material 4).

### 2.2 Rotifer feeding

Rotifer cultures were established using natural seawater (SeaW) sourced from the coastal regions of Hainan, China, with a salinity adjusted to 30 ± 2 ‰. This SeaW was sterilized via autoclaving prior to use in both rotifer and microalgal cultures. The rotifers were continuously fed with *Chlorella* sp. NeZha at cell densities ranging from 10 to 20 × 10^6^ cells/mL, which aligns with densities recommended for intensive rotifer cultures (13). Environmental conditions were maintained at a constant room temperature of 25 ± 1°C with an illumination level of 100 μmol/m^2^/s.

### 2.3 Microalgal cultivation

For the experimental setup, each culture medium in a 500 mL flask, with an active culture volume of 300 mL. The salinity of each algal culture medium BG11 was adjusted to salinity of 0 ‰ (FreshW), 30 ‰ (SeaW), and 45 ‰ (HiSal) using sodium chloride (NaCl). At the beginning of the experiment, the initial cell density of the algal suspension was measured and an equal volume of algal suspension was added to each medium to achieve an initial density of approximately 1 × 10^5^ cells/mL.

The culture media were placed in a controlled environment chamber without air circulation. The light cycle was set at 12 h light to 12 h dark, with a light intensity of 100 μmol/m^2^/s, and the temperature maintained at 25 ± 1°C. Cell counts were conducted daily using an optical microscope and a hemocytometer. All experimental vessels were sterilized at 121°C under high pressure. Each experimental group included at least three replicate samples to ensure the reliability of the results.

### 2.4 Batch culture of microalgae

The experimental cultivation of *Chlorella* sp. NeZha was conducted in a 400 L tubular photoreactor located outdoors. The BG11 medium, modified to a salinity of 3‰, was employed as the growth medium. Initial cell density was set at 3.5 × 10^6^ cells/mL. Throughout the 6-day culture period, the system was maintained between temperatures of 22.5 and 25.6°C, with day light densities ranging from 300 to 1,250 μmol/m^2^/s, ensuring adequate light exposure for photosynthesis and growth. The growth rate, represented as μ (day^−1^), derived from cell counts as μ =  (ln(*N*_t_)-ln(*N*_0_))/(*t* − *t*_0_), where *N*_t_ and *N*_0_ are the cell concentrations at time *t* and *t*_0_, respectively, and (*t* − t_0_) gives the duration (day) elapsed between the measurements. The production rate calculated from weight, production rate =  (W_t_ − W_0_)/(*t* − t_0_) V, where W_t_ and W_0_ are the dry weight of alga cell at time *t* and *t*_0_, respectively, and (*t* − *t*_0_) gives the duration (day) elapsed between the measurements, V, 400 L.

### 2.5 Bioactive materials in *Chlorella* sp. NeZha cells

The content of Chl a, proteins, and lipids was quantitatively analyzed using colorimetric and chromatographic techniques. Algal cells were collected by centrifugation at 7,000 rpm for 10 min, and freeze-dried for bioactive substance content determination.

#### 2.5.1 The pigment content

Algae cells (10 mg) were extracted overnight at 4°C with 95% ethanol. The samples then centrifuged at 5,000 rpm for 10 min. Optical densities at 470, 649, and 665 nm of the supernatant was detected. The content of Chl a, Chl b, and carotenoids were calculated using the following formulas:

Chla(mg/L)=13.95A665−6.88A649

Chlb(mg/L)=24.96A649−7.32A665

Car(mg/L)=(1000A470−205Chla−1148Chlb)/245

#### 2.5.2 Total lipids content

100 mg of algae was extracted with 9.5 mL of a mixed solution (chloroform: methanol: distilled water = 1: 2: 0.8) for 10 min, repeated for three times. The supernatant was collected after centrifugation. 7.5 mL of distilled water and chloroform were added to the supernatant, the ratio of mixed solution was adjusted to chloroform: methanol: distilled water = 1: 1: 0.9. The organic phases were collected into a pre-weighted glass tube, dried under nitrogen gas (N_2_), and calculate the total lipids content gravimetrically.

#### 2.5.3 Protein content

Total proteins were quantified using the BCA protein assay (PC0020, Solarbio). 20 μL of the samples was added to a 96-well plate, and 200 μL of BCA working solution was added to each well at 37°C for 30 min. The optical density at a wavelength of 562 nm was detected, and the protein concentration of the sample was calculated from the standard curve.

#### 2.5.4 Carbohydrate content

The phenol-sulfuric acid method was applied to quantify the carbohydrate content. Samples (10 mg) were mixed with 2 mL deionized water and 1 mL 6% (W/V) phenol, then added 5 mL sulfuric acid for 30 min. The absorbance of the mixed solution was detected under the wavelength of 490 nm, and the concentration of carbohydrate was obtained with the standard curve.

### 2.6 Microalgae cultivation in *Artemia* wastewater

The *Artemia* cultivation facility is located near the coast of Hainan Island, China, where *Artemia* are grown in brackish water within indoor tanks, maintained at 25°C. Effluent from the final *Artemia* production stage is settled to remove large particulates and subsequently filtered through 47 mm Whatman GF/F filters prior to its use in microalgal cultivation. The effluent characteristics recorded were biochemical oxygen demand (BOD) between 25 and 35 mg/L, chemical oxygen demand (COD) between 65 and 80 mg/L, and a pH ranging from 6.5 to 7.2. Replicate experiments were conducted with an initial algal concentration of 3.5 × 10^6^ cells/mL in 5 L plastic tanks. Over a 6-day period, the cultures were continuously aerated, and cellular concentration and growth were assessed daily using a Hemocytometer.

### 2.7 Statistical analysis

To ensure robustness and reliability, three independent biological replicate cultivations were conducted. Statistical evaluation of the obtained results employed the Generalized Linear Model (*p* < 0.001) and Tukey’s Honestly Significant Differences (HSD) multiple comparison test within the Statistical Package for the Social Sciences (SPSS) software version 16.0.

## 3 Results

### 3.1 Microalgae strain verification and rotifers feeding

**Figure 1:**
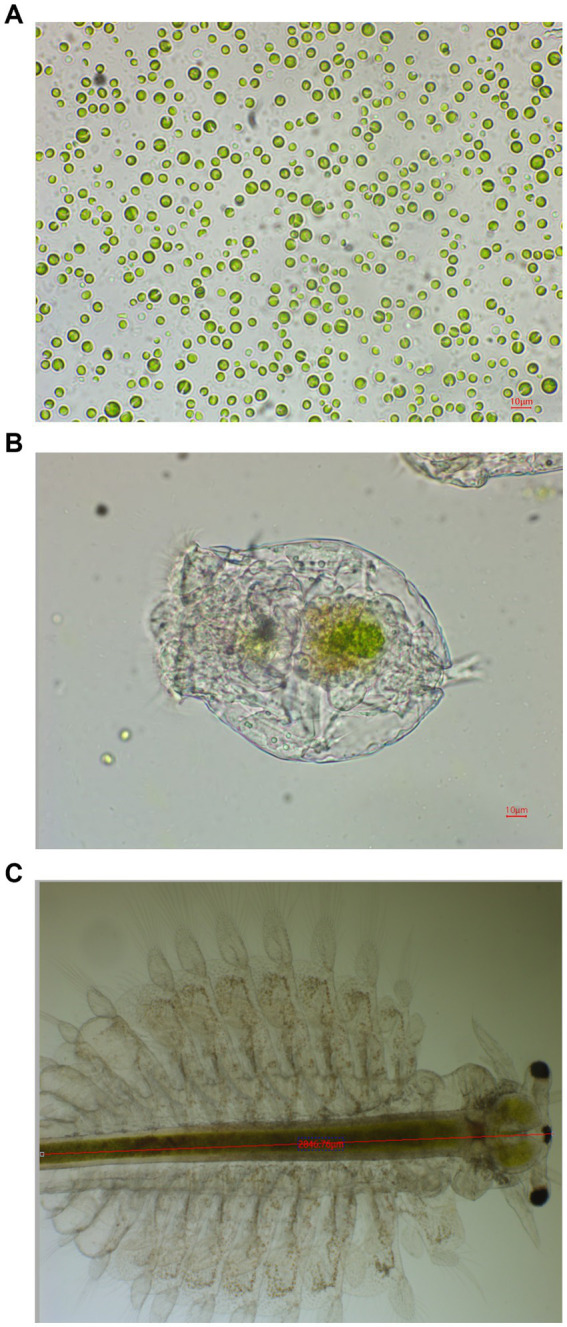
Representative microscopic photos of *Chlorella* sp. NeZha **(A)**, a rotifer **(B)**, and a *Artemia* **(C)** with *Chlorella* cells in their gastrointestinal tracts.

Microalgae observed under the light microscope, the microalgae (Figure 1A) have the same characteristics as *Chlorella* sp., with a circular or oval shape with a diameter ranging from 4 to 12 μm. According to the observation, the microalgae were green in color, indicating that they belong to the Chlorophyta division (31). Our 18S rDNA and ITS sequencing results verified this conclusion (Supplementary material).

Our detailed observations showed that within the gastrointestinal tracts of both rotifers and *Artemia* (Figures 1B,C), there was a significant presence of *Chlorella* sp. NeZha, identifiable as green granules. This was particularly noticeable 1 h after these zooplankton were fed live microalgae, indicating rapid ingestion and digestion. Initially, the culture medium exhibited a green hue due to the presence of the microalgae, but it became clear shortly after feeding, suggesting effective consumption and utilization of the algae by the rotifers and *Artemia*.

Continued monitoring over several days revealed that the rotifers and *Artemia* not only survived but also thrived, with populations increasing noticeably. These outcomes demonstrate that *Chlorella* sp. NeZha algae are non-toxic to rotifers and *Artemia* and are of a suitable size for them to ingest, supporting the viability of NeZha algae as a potential live feed. This suitability could make *Chlorella* sp. NeZha algae an excellent candidate for sustainable aquaculture practices, where effective and non-toxic feed options are critical for the health and proliferation of cultured species.

Further detailed investigation into the growth and quality of rotifers and *Artemia* will undoubtedly confirm the potential of *Chlorella* sp. NeZha as a feed for these organisms.

### 3.2 Growth under different salinities, revealing adaptive patterns

**Figure 2:**
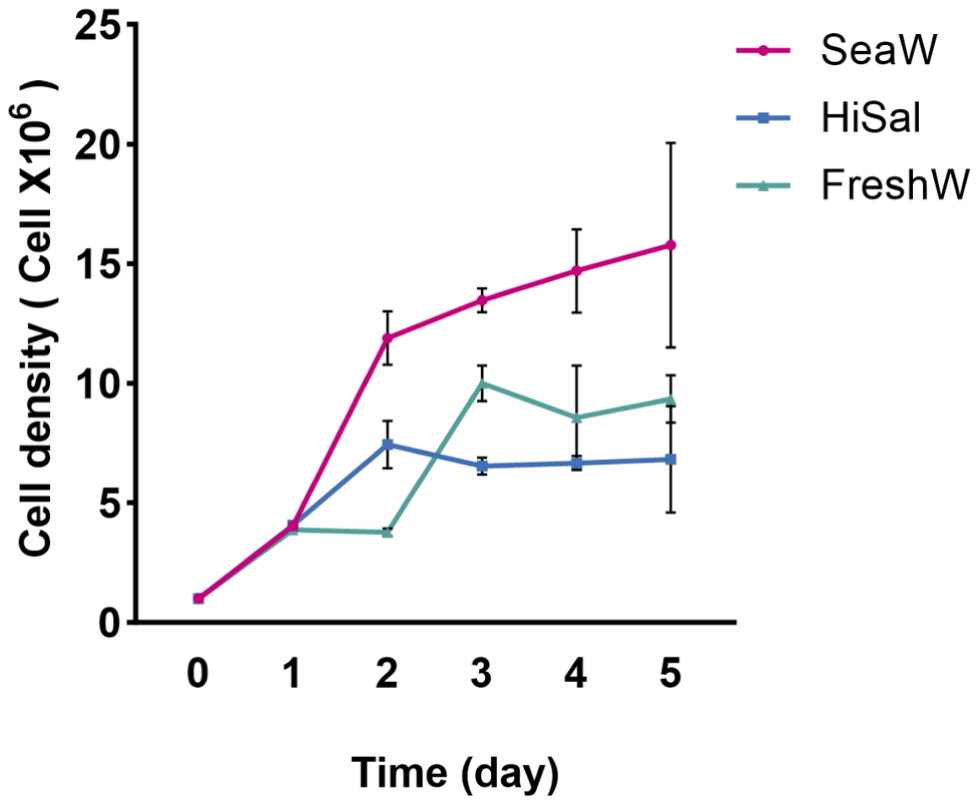
*Chlorella* sp. NeZha growth under different salinities, SeaW with 30‰, HiSal with 45‰, and FreshW with 0‰ salt added in the BG11, within 300 mL culture medium in 500 mL flasks Cell number 1 × 10^6^ cells/mL.

The growth of *Chlorella* sp. NeZha under different salinity conditions revealed distinct patterns across the experimental setups. In the medium with 30‰ salt concentration (SeaW), the algae displayed the most robust growth, with cell numbers steadily rising from an initial 1 × 10^6^ cells/mL to approximately 15.775 × 10^6^ cells/mL by the sixth day. The sharp increase was particularly pronounced in the early phase of the experiment, demonstrating a favorable response to this salinity level (Figure 2).

Conversely, the 45‰ salt medium (HiSal) initially supported a comparable growth rate, but the cell count plateaued around day 3, suggesting that higher salt concentrations may start to inhibit growth (Figure 2). This condition peaked at a lower cell density than the 30‰ salt medium (SeaW), indicating a less optimal environment for sustained growth.

The control group FreshW, cultured in standard BG11 medium without additional salinity, showed a different growth trajectory (Figure 2). Although it started similarly to the other groups, its growth rate varied more and generally remained lower than in the 30‰ salt medium (SeaW). By day 6, the control group reached about 9.342 × 10^6^ cells/mL, displaying less stability compared to the modified salinity conditions.

When comparing the growth in 30‰ salt medium (SeaW) against the 45‰ salt medium (HiSal), it becomes evident that a lower salinity within this range is more conducive to *Chlorella* sp. NeZha algae proliferation. The algae not only grew faster but also sustained higher densities over time in the 30‰ salt medium (SeaW).

Similarly, the comparison between the 30‰ salt medium (SeaW) and the control highlights the beneficial effects of moderate salinity enhancement. The algae in the 30 ‰ salt medium (SeaW) consistently outperformed those in the control across all days, underscoring the positive impact of salt on growth dynamics.

In contrast, algae in the 45 ‰ salt medium (HiSal) did not fare as well as those in the control beyond the initial growth spurt, illustrating potential salinity stress at higher concentrations. This observation is crucial for understanding the upper salinity limits that *Chlorella* sp. NeZha algae can tolerate before growth is adversely affected.

Overall, the experiment demonstrates that *Chlorella* sp. NeZha algae thrive best in a BG11 medium augmented with 30‰ salt as the salinity of seawater (SeaW). This condition optimizes growth compared to both higher salinity and unsupplemented control conditions. Furthermore, the ability of *Chlorella* sp. NeZha algae to adapt to varying salinity levels highlights its robustness and potential for diverse aquacultural applications. These insights are significant, suggesting that careful adjustment of salinity levels can enhance algae cultivation efficiency.

### 3.3 Bioactive materials in *Chlorella* sp. NeZha

**Figure 3:**
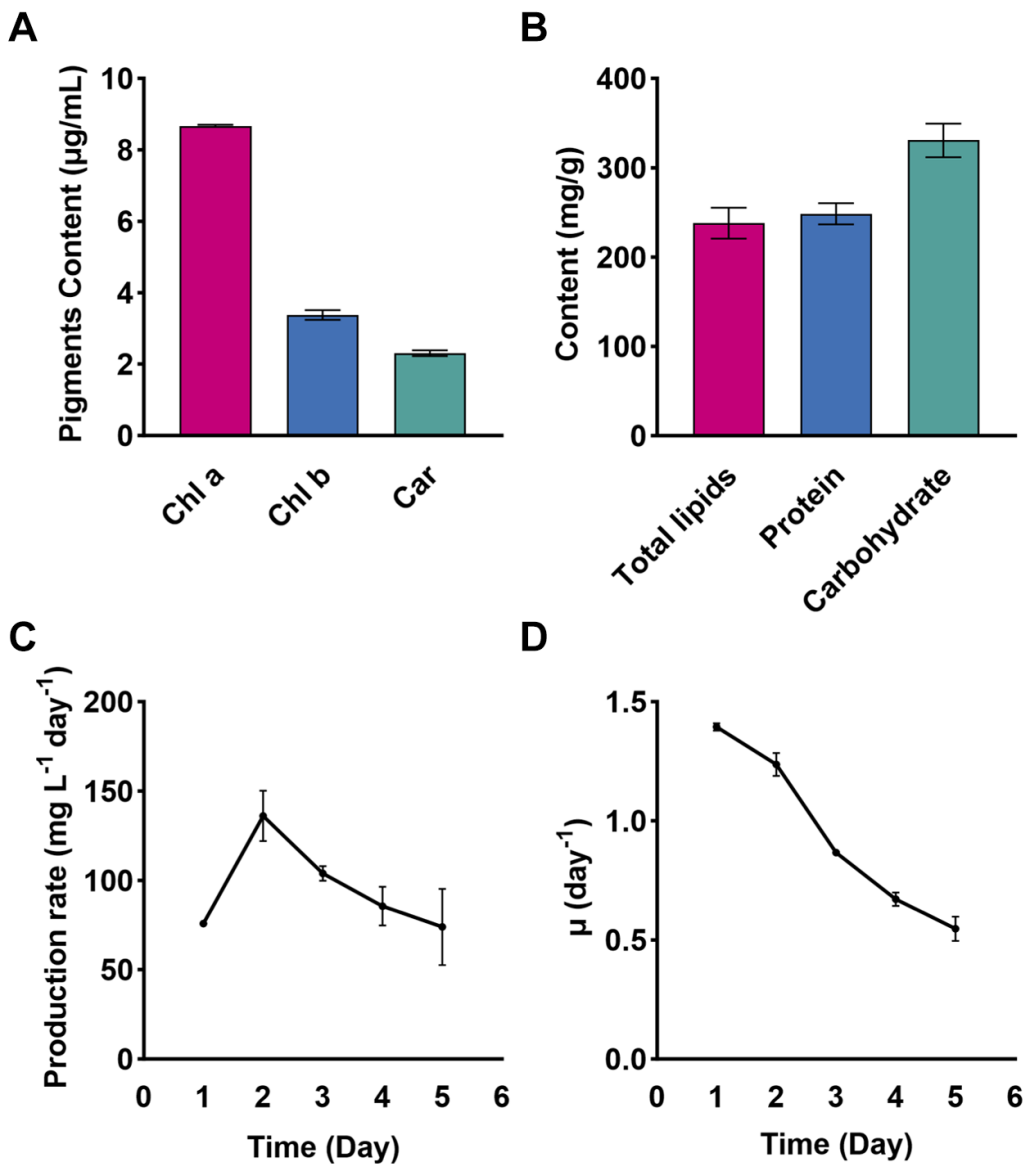
Pigments Chl a, Chl b, and Car (Chlorophyll a, b, and carotenoids) in *Chlorella* sp. NeZha **(A)**; total lipids, protein, and carbohydrate contents in *Chlorella* sp. NeZha **(B)**; production rate **(C)**, and special growth rate **(D)**. Data were collected under culture in 400 L bioreactors, with 30‰ salinity in an outdoor bioreactor (Supplementary material 4).

On day 6 of cultivation in a 400 L bioreactor (Supplementary material), *Chlorella* sp. NeZha exhibited the following concentrations of bioactive substances (mg/g dry weight): carbohydrates at 332.12 were the most abundant, followed by proteins at 198.73, lipids at 122.99, Chl a at 34.67, Chl b at 13.49, and carotenoids at 9.20 (Figures 3A,B).

Comparative analysis of Chl a and b reveals that Chl a concentrations were substantially higher, which typically indicates a greater role in capturing light energy for photosynthesis. The ratio of Chl a to Chl b further underscores the algae’s adaptation to its light environment, with Chl a being more predominant (Figure 3A).

Further comparing the pigments, Chl a was about 2.6 times higher than Chl b and nearly 3.8 times more than carotenoids. This distribution suggests efficient energy capture and protective mechanisms against photodamage, as carotenoids play a crucial role in protecting photosynthetic systems from oxidative stress (Figure 3A).

Among the macromolecules, carbohydrates were the highest, indicating significant energy storage, followed by proteins, which are essential for growth and repair, and lipids, which are crucial for membrane formation and energy storage, and have important implications for biofuel production (Figure 3B).

The production rate of the substance peaks at approximately 150 mg L^−1^ day^−1^ on Day 2, then gradually declines to around 70 mg L^−1^ day^−1^ by Day 6 (Figure 3C). The specific growth rate starts at 1.5 day^−1^ on Day 1 and continuously declines to about 0.3 day^−1^ by Day 6 (Figure 3D). Both graphs indicate an initial high rate of production and growth, followed by a steady decline over the 6-day observation period. The production rate peaks early and then gradually decreases, while the specific growth rate shows a continuous decline from the beginning to the end of the period.

Overall, the data indicate robust growth and metabolic activity in *Chlorella* sp. NeZha, with a balanced accumulation of pigments and macromolecules that highlight its potential for bioenergy and nutritional applications.

### 3.4 Microalgal growth in *Artemia* wastewater

The growth of *Chlorella* sp. NeZha in *Artemia* wastewater was monitored preliminary over a six-day period. The overall trend indicates that *Chlorella* sp. NeZha algae thrived well in wastewater, with a marked increase in cell concentration each day. Initially, the cell density started at approximately 3.38 million cells/mL on day 1, escalating steadily to about 8.39 million cells/mL by day 2, and reaching 12.98 million cells/mL on day 3. The growth continued to 16.58 million cells/mL on day 4 and 21.44 million cells/mL by day 5. By day 6, the cell concentration slightly increased to 22.51 million cells/mL, suggesting that the algae nearly reached a plateau phase. This rapid growth and high cell density achievement in a relatively short time highlight *Chlorella* sp. NeZha algae’s capability to efficiently utilize the nutrients available in *Artemia* wastewater.

This rapid growth and high cell density achievement highlight *Chlorella* sp. NeZha algae’s potential for integration into *Artemia* culturing systems, suggesting the feasibility of directly feeding live *Chlorella* sp. NeZha algae in *Artemia* aquaculture ponds.

## 4 Discussion

### 4.1 A salt-tolerant algae strain with robust environmental adaptability

The experimental cultivation of *Chlorella* sp. NeZha under varying salinity conditions revealed distinct growth patterns, underscoring the importance of optimizing environmental parameters for microalgal cultivation. Our findings showed that the 30‰ salt concentration medium supported the most robust growth, with cell densities rising from 1 × 10^5^ cells/mL to approximately 15.775 × 10^6^ cells/mL over 6 days. This rapid proliferation highlights the favorable response of *Chlorella* sp. NeZha algae to moderate salinity levels, aligning with previous research indicating that moderate salinity can enhance microalgal growth and biomass productivity (5). Conversely, the higher salinity medium (45‰) initially supported growth but plateaued by day 3, indicating that elevated salt concentrations may inhibit sustained algal proliferation. This observation is consistent with the findings of Church et al. (28), who reported that while increased salinity can enhance lipid accumulation, it may also reduce growth rates. The control group, cultivated without additional salinity, showed lower and more variable growth, reaching about 9.342 × 10^6^ cells/mL by day 6, further emphasizing the growth benefits of moderate salinity. The results of our study are in line with those of Almutairi et al. (32), who demonstrated that *Chlorella vulgaris* adapted to high salinity can achieve significant biomass yields. However, our findings suggest that a moderate salinity level (30‰) is optimal for *Chlorella* sp. NeZha, providing a balance between growth and potential stress-induced lipid accumulation. Similar conclusions were drawn by Gour et al. (33), who found that stepwise salinity stress can optimize both growth and lipid productivity in microalgae. Furthermore, the use of NaCl to adjust salinity in our experimental setup parallels the study by Ghobrini et al. (34), which explored heterotrophic cultivation of *Chlorella vulgaris* using saline wastewater. Their findings support the feasibility of using saline conditions to cultivate microalgae for biomass production, a potential strategy for integrating wastewater management with biofuel production. Additionally, our results contribute to the broader understanding of microalgal responses to environmental stress, as discussed by Shetty et al. (35), who reviewed the salinity stress responses and adaptation mechanisms in eukaryotic green microalgae.

The optimal salinity condition of 30‰ for *Chlorella* sp. NeZha enhances growth and biomass accumulation, highlighting its potential for efficient cultivation in saline environments. These findings support the integration of *Chlorella* sp. NeZha into sustainable aquaculture and biofuel production systems, leveraging its resilience and adaptability to moderate salinity conditions. Further research should explore the long-term effects of salinity stress on lipid accumulation and overall biomass productivity to fully harness the potential of *Chlorella* sp. NeZha algae in various industrial applications.

### 4.2 Comparison of Chl a content

Chlorophyll a content in microalgae is a critical indicator of their photosynthetic capacity and has significant implications for biotechnological applications. In our study, *Chlorella* sp. NeZha exhibited remarkably high Chl a content under room temperature conditions with BG11 medium supplemented with 30‰ salt in an outdoor 500 L bioreactor. Specifically, we observed a total chlorophyll content of 48.14 mg/g, 34.73 pg./cell, and 12.1 μg/mL, while the Chl a content was 34.25 mg/g, 25 pg./cell, and 8.6 μg/mL.

When compared to other microalgae (Table 1), *Chlorella* sp. NeZha stands out for its high chlorophyll production. For instance, Cheirsilp and Torpee (19) reported Chl a content of 22.98–24.15 mg/g in marine *Chlorella* sp. under mixotrophic conditions, which is significantly lower than the 34.25 mg/g observed in our study. Similarly, Cakmak et al. (16) found Chl a content ranging from 0.03 to 0.035 pg./cell in *Chlamydomonas reinhardtii* strains under nutrient deprivation, and Ferreira et al. (21) reported 1.6 pg./cell in *Scenedesmus dimorphus* under low light intensity, both of which are considerably lower than the 25 pg./cell measured for *Chlorella* sp. NeZha.

**Table 1:** A summary of microalgal Chl a (Chl a) and total chlorophyll with different measurement units.

Microalgae species	Chlorophyll contents	Treatments	References
*Ankistrodesmus falcatus*	5.19–17.78 μg/mL	Culture medium	George et al. (22)
*Ankistrodesmus falcatus* (Chl a)	4.47–13.87 μg/mL	Culture medium	George et al. (22)
*Cheatoceros* sp.	15.49–23.65 mg/g	Photo, hetero-, mixotrophic	Cheirsilp and Torpee (19)
*Chlamydmonas reinhardtii* (Chl a)	2.56 pg./cell	Nitrogen	Dean et al. (17)
*Chlamydmonas reinhardtii* CC124, 125	0.03–0.035 pg./cell	N -,S-	Cakmak et al. (16)
*Chlorella fusca*	11–45 μg/mL	N-, S-starvation	Jerez et al. (36)
*Chlorella pyrenoidosa*	15–29 mg/g	N-,P-,Fe-	Fan et al. (24)
*Chlorella* sp. (NIVA CHL-137)	7.12 mg/g	axenic, non-axenic	Halfhide et al. (37)
*Chlorella* sp. L1 (Chl a)	10–15 μg/mL	LL,ML,HL	He et al. (38)
*Chlorella* sp. Freshwater	18.78–22.1 mg/g	LL,ML,HL	Cheirsilp and Torpee (19)
*Chlorella* sp. Marine	22.98–24.15 mg/g	Glucose	Cheirsilp and Torpee (19)
*Chlorella* sp. NeZha	48.14 mg/g, 34.73 pg./cell, 12.1 μg/mL	Room temperature	This study
*Chlorella* sp. NeZha (Chl a)	34.25 mg/g 25 pg./cell, 8.6 μg/mL	BG11 + salt 3%	This study
*Chlorella vulgaris*	0.5–2.4%	Nitrogens	Lv et al. (39)
*Dunaliella salina*	2.6–38.5 mg/g	Photo, hetero-, mixotrophic	Gim et al. (23)
*Isochrysis galbana*	4.1–32.3 mg/g	Photo, hetero-, mixotrophic	Gim et al. (23)
*Microcystis aeruginosa* (Chl a)	11.86 mg/g	Phosphorous 1,4,10 mg/L	Chen et al. (20)
*Nannochloropsis oculata*	3.7–41.9 mg/g	Photo, hetero-, mixotrophic	Gim et al. (23)
*Nannochloropsis* sp.	22.49–27.05 mg/g	LL,ML,HL	Cheirsilp and Torpee (19)
*Pavlova lutheri* (Chl a)	0.47 pg./cell	Temp, Light	Carvalho et al. (18)
*Pavlova viridis*	0.4 pg./cell	Zn,Cu	Li et al. (40)
*Scenedesmus dimorphus*	1.6 pg./cell	N-, LL	Ferreira et al. (21)
*Scenedesmus obliquus* (Chl a)	16.23 mg/g	15,25C	Chen et al. (20)
*Scenedesmus subspicatus* (Chl a)	0.617 pg./cell	Nitrogen	Dean et al. (17)

Our findings also compare favorably with high-performing species such as *Scenedesmus obliquus*, which exhibited Chl a content of 16.23 mg/g under specific phosphorus and temperature conditions (20). *Chlorella* sp. NeZha’s Chl a content of 34.25 mg/g indicates superior efficiency in chlorophyll production under saline conditions. Gim et al. (23) reported Chl a content ranging from 2.6 to 38.5 mg/g in *Dunaliella salina*, which, while comparable, is generally lower than our findings, further highlighting the potential of *Chlorella* sp. NeZha.

### 4.3 Influence of cultivation conditions on Chl a content

Chlorophyll a content in microalgae is significantly influenced by various culture conditions, including nutrient availability, light intensity, and temperature. Our study underscores the importance of optimizing these conditions to maximize chlorophyll production.

For instance, nutrient availability, particularly nitrogen and phosphorus, plays a crucial role. Cakmak et al. (16) and Dean et al. (17) highlighted how nutrient deprivation can lead to significant reductions in Chl a content. Conversely, nutrient-rich conditions can enhance chlorophyll synthesis, as demonstrated by Cheirsilp and Torpee (19), who reported higher Chl a content under mixotrophic conditions.

Light intensity is another critical factor. Ferreira et al. (21) and Gim et al. (23) showed that increased light intensity boosts Chl a content in various microalgae species. Similarly, our study’s outdoor bioreactor conditions, with adequate light exposure, likely contributed to the high chlorophyll levels observed in *Chlorella* sp. NeZha. One of the key advantages of our study is the achievement of high Chl a content under a single cultivation condition. Using BG11 medium with 3% salt (30 ‰) in an outdoor bioreactor, *Chlorella* sp. NeZha demonstrated robust chlorophyll synthesis, which is promising for large-scale applications. However, the study also has limitations. We did not explore multiple cultivation methods that could potentially enhance chlorophyll production further. Future research should investigate various nutrient conditions, light intensities, and temperatures to optimize chlorophyll content. Additionally, scaling up the bioreactor conditions to industrial levels could provide more insights into the practical applications of *Chlorella* sp. NeZha.

In conclusion, *Chlorella* sp. NeZha shows exceptional potential for high Chl a production under saline conditions, outperforming many previously studied microalgae. Our findings provide a strong foundation for further research aimed at optimizing cultivation conditions to maximize chlorophyll content, with significant implications for biofuel production and other biotechnological applications. Future studies should focus on exploring diverse cultivation strategies to achieve even higher chlorophyll yields and assess the scalability of these methods for industrial use.

### 4.4 Rotifer feeding

The phytoremediation of aquaculture wastewater with microalgae has great potential due to its high nutrient removal efficiency and low cost (41). Our study’s findings demonstrated that *Chlorella* sp. NeZha effectively supports the growth and survival of rotifers in aquaculture, positioning it as a viable candidate for sustainable feed. The rapid ingestion and digestion of *Chlorella* sp. NeZha by rotifers, as indicated by the presence of green granules in their gastrointestinal tracts shortly after feeding, highlights its suitability and non-toxicity. This aligns with previous studies that have shown the benefits of using nutrient-rich microalgae in aquaculture. For instance, Rahman et al. (13) demonstrated that various microalgae species, including *Chlorella*, significantly enhance rotifer growth rates. Similarly, Ferreira et al. (11) noted improved survival and nutritional quality of rotifers when fed high-quality microalgae. The increase in rotifer populations observed in our study further supports the potential of *Chlorella* sp. NeZha as an effective feed. This result is consistent with findings by Lucía-Pavón et al. (12), who reported that live *Chlorella vulgaris* significantly boosts rotifer population growth compared to other forms. Moreover, the sustainability and non-toxic nature of *Chlorella* sp. NeZha as a feed are crucial advantages, as highlighted by Aly et al. (3), who emphasized the importance of safe and eco-friendly alternatives in aquaculture to avoid the drawbacks of traditional chemical treatments. The successful use of *Chlorella* sp. NeZha in our study also contributes to the broader discourse on the role of microalgae in aquaculture sustainability. Tham et al. (15) reviewed the potential of microalgae as substitutes for fish oil and fish meal in aquafeeds, noting their high lipid, protein, and carbohydrate content essential for fish growth. Our findings align with this perspective, demonstrating that *Chlorella* sp. NeZha can provide a nutrient-rich, sustainable feed option that supports not only rotifer growth but also potentially the health and development of higher trophic levels in aquaculture systems. Furthermore, the use of *Chlorella* sp. NeZha may offer environmental benefits through its application in bioremediation and water quality improvement, as suggested by Aly et al. (3). The presence of *Chlorella* in the culture medium initially imparted a green hue, which cleared after rotifer feeding, indicating effective consumption and potential nutrient recycling capabilities. This aspect aligns with the eco-friendly attributes of microalgae-based systems, contributing to improved water quality and sustainable aquaculture practices.

Thus, the integration of *Chlorella* sp. NeZha into rotifer cultures demonstrates its effectiveness as a feed, promoting growth and survival while offering environmental sustainability. These findings, supported by previous research, highlight the potential of *Chlorella* sp. NeZha as a valuable component in the development of sustainable aquaculture feeds, contributing to the broader goals of ecological balance and resource efficiency in aquaculture operations. There are some advantages and significance of *Chlorella* sp. NeZha as feed for *Artemia*: (1) it can be fed to *Artemia* live, eliminating the costly and labor-intensive steps of centrifugation and drying. (2) It has high chlorophyll and polysaccharide content, which is expected to enhance Artemia’s disease resistance and survival rate. (3) Although cultivation conditions have not yet been optimized, its phenotypic performance in seawater, brackish water, and wastewater suggests significant potential for improvement. These points demonstrate that *Chlorella* sp. NeZha has great potential for application as *Artemia* feed in aquaculture.

Further research should explore its application across different aquaculture species and environmental conditions to fully harness its benefits.

### 4.5 Microalgal culitvation in *Artemia* wastewater

The results of this study demonstrate the viability of using *Chlorella* sp. NeZha for the treatment and utilization of *Artemia* wastewater in microalgal cultivation. The effluent from *Artemia* production, characterized by moderate levels of biochemical oxygen demand (BOD) and chemical oxygen demand (COD), provided a nutrient-rich medium for the growth of NeZha algae. Over a 6-day cultivation period, *Chlorella* sp. NeZha exhibited a significant increase in cell density, reaching a peak concentration of 22.51 million cells/mL, indicating efficient nutrient utilization and robust growth in the wastewater environment. These findings are consistent with previous studies highlighting the potential of microalgae in wastewater treatment and biomass production. For instance, Bhuyar et al. (31) demonstrated enhanced growth of *Chlorella vulgaris* in wastewater effluent from tilapia culture ponds, achieving significant biomass yields suitable for bioethanol production. Similarly, Halfhide et al. (37) reported successful cultivation of *Chlorella* sp. in aquaculture wastewater, noting that non-axenic conditions did not hinder biomass development and even enhanced lipid content.

Furthermore, the observed rapid growth of *Chlorella* sp. NeZha in *Artemia* wastewater aligns with the findings of Daneshvar et al. (29), who investigated the feasibility of cultivating *Chlorella vulgaris* in a mixture of pulp and aquaculture effluents. The study showed high dry algal weight and efficient nutrient removal, underscoring the potential of microalgae to thrive in mixed wastewater environments. Our results also parallel those of Stamenković et al. (42), who found that desmid strains could effectively remediate moderately saline aquaculture wastewater, highlighting the adaptability of different microalgal species to varying wastewater conditions.

Incorporating *Chlorella* sp. NeZha into *Artemia* culturing systems offers multiple benefits, including nutrient recycling and potential feed applications. The high cell density achieved in our study suggests that *Chlorella* sp. NeZha algae can be directly utilized as live feed in *Artemia* aquaculture ponds, supporting sustainable aquaculture practices. This approach not only enhances the ecological efficiency of aquaculture operations but also reduces the reliance on conventional feeds, contributing to a circular bioeconomy as discussed by Tham et al. (15).

The successful cultivation of *Chlorella* sp. NeZha in *Artemia* wastewater demonstrates its potential for integration into sustainable aquaculture systems. This study provides valuable insights into the practical applications of microalgae in wastewater treatment and biomass production, paving the way for further research and optimization in diverse aquaculture settings.

## 5 Conclusion

The recent isolation of *Chlorella* sp. *NeZha* from a balcony environment has uncovered a strain with remarkable properties. Our study highlights the robust environmental adaptability and high salt tolerance of *Chlorella* sp. NeZha, making it a promising candidate for sustainable aquaculture and biofuel production. The optimal growth observed at a 30‰ salinity level underscores its ability to efficiently utilize nutrients and thrive in saline conditions. Furthermore, the successful integration of *Chlorella* sp. NeZha algae into *Artemia* wastewater systems demonstrates its potential for nutrient recycling and as a viable feed option, supporting ecological balance and resource efficiency. These findings pave the way for further research into optimizing cultivation conditions and exploring diverse industrial applications of *Chlorella* sp. NeZha, contributing to sustainable and eco-friendly practices in various sectors.

## Data Availability

The original contributions presented in the study are included in the article/Supplementary material; further inquiries can be directed to the corresponding author.
