# Supplementary material for: Adaptability and nutritional analysis of a newly isolated Chlorella sp. NeZha in brackish and marine environments with potential bioeconomic impacts
Source: Front Nutr. 2024 Aug 14;11:1460675. doi: 10.3389/fnut.2024.1460675 (PMC11349555; doi:10.3389/fnut.2024.1460675)

**Supplementary data**

**1, Primers used in this study**

18S rDNA primers (Forward 5'-CCAACCTGGTTGATCCTGCCAGTA-3'; reverse 5'- CCTTGTTAACGACTTCACCTTCCTCT-3')

ITS rDNA primers (Forward 5'-TCCGTAGGTGAACCTGCGG-3'; reverse 5'- TCCTCCGCTTATTGATATGC-3')

**Supplementary data**

**2, Partial sequence of 18S rDNA *Chlorellla* sp. NeZha**:

5'-CCAAGATTTAAAGGCCCATTGGCATGGTCTAAGTATAAACTGCTTTATACTGTGAAACTGCGAATGGCTCATTAAATCAGTTATAGTTTATTTGATGGTACCTTACTACCGGATAACCGTAGTAATTCTAGAGCTAATACGTGCGTAAACCCCGACTCCTGGAAGGGGCGTATTTATTAGATTTAAGGCCGACCCGGCTCTGCCGGTCTCGCGGTGAATCATGATAACTTCACGAATCGCATGGCCTTGTGCCGGCGATGTTTCATTCAAATTTCTGCCCTATCAACTTTCGATGGTAGGATAGAGGCCTACCATGGTGGTAACGGGTGACGGAGGATTAGGGTTCGATTCCGGAGAGGGAGCCTGAGAAACGGCTACCACATCCAAGGAAGGCAGCAGGCGCGCAAATTACCCAATCCTGACACAGGGAGGTAGTGACAATAAATAACAATACCGGGCCTTTTCAGGTCTGGTAATTGGAATGAGTACAATCTAAACCCCTTAACGAGGATCAATTGGAGGGCAAGTCTGGTGCCAGCAGCCGCGGTAATTCCAGCTCCAATAGCGTATATTTAAGTTGCTGCAGTTAAAAAGCTCGTAGTTGGATTTCGGGCGGGGCCTGCCGGTCCGCCGTTTCGGTGTGCACTGGCCGGGCCCGCCTTGTTGCCGGGGACGGGCTCCTGGGCTTCACTGTCCGGGACTCGGAGTCGGCGCTGTTACTTTGAGTAAATTAGAGTGTTCAAAGCAGGCCTACGCTCTGAATACATTAGCATGGAATAACACGATAGGACTCTGGCCTATCCTGTTGGTCTGTAGGACCGGAGTAATGATTAAGAGGGACAGTCGGGGGCATTCGTATTTCATTGTCAGAGGTGAAATTCTTGGATTTATGAAGACGAACTACTGCGAAAGCATTTGCCAAGGATGTTTTCATTAATCAGAACGAAAGTTGGGGGCTCGAGACGATTAGATACGTCTAGTCTTCAACATAAACGATGCCGACTAGGGATCGGCGATGTTTCTTCGATGACTTCGCGGCACCTTAATGAAGAAATCAAA -3'

**Supplementary data**

**3, Partial sequence of ITS rDNA *Chlorellla* sp. NeZha:**

5'-GAATGCCAAATCTAACGTGATGACGATCACACCGGTGACCACACAAACCCCCCTTGCCTCACCCCCAAGGGGCGCCAGTCCCCTGGCCCCGGCGCTCAGCCGCGGTGTCCAGGTCTGGCGGGGTGGGGGCGGAGCCTCTGGCATGCCTATACACCAGTGCTAACCACTGTCAAAACCAAACTCTGAAGTTTTGATTGCTAGTAACTGGCAATCCTAACCAAAGACAACTCTCAACAACGGATATCTTGGCTCTCGCAACGATGAAGAACGCAGCGAAATGCGATACGTAGTGTGAATTGCAGAATTCCGTGAACCATCTAATCTTTGAACGCATATTGCGCTCGAGCCCTCGGGCAAGAGCATGTCTGCCTCAGCGTCGGTTTACACCCTCACCCCTCTTTCCTTTTGGATCGCAGGTTAGCTTATCAGCTGGCCCTAGGGGTGGATCTGGCTTTCCCAATCCTTTCTGGGTTGGGTTGGCTGAAGTGTAGAGGCTTAATCAAGGACCCGATATGGGCTTCAACTGGATAGGTAGCAACGGCTTCTGCCGACTACACGAAGTTGGAGCTTGTGGACTTTGATAGGAGCCAAGCAGGAAACGTGCTTGCACGTTCTAAACTTTCGACCTGAGCTCAGGCAAGGCTACCCGCTGAACTTAAGCATATCAATAAGCGGAGG-3'

**Supplementary data**

**4. Photo of outdoor bioreactor**


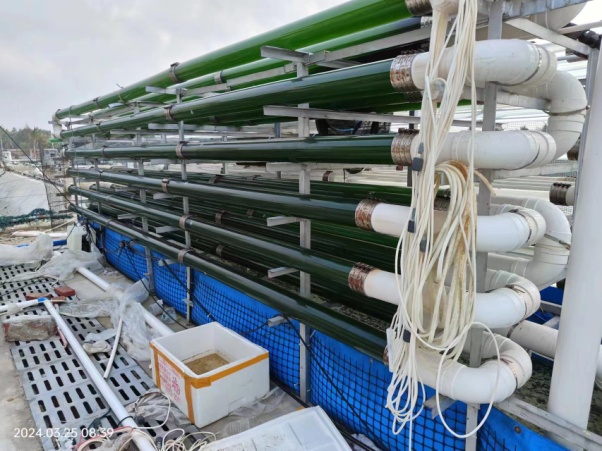

Supplement: Supplementary file 1 [file Data_Sheet_1.docx]
